# Graphene-Derivatized Silica Composite as Solid-Phase Extraction Sorbent Combined with GC–MS/MS for the Determination of Polycyclic Musks in Aqueous Samples

**DOI:** 10.3390/molecules23020318

**Published:** 2018-02-02

**Authors:** Cheng Li, Jiayi Chen, Yan Chen, Jihua Wang, Hua Ping, Anxiang Lu

**Affiliations:** 1 Beijing Research Center for Agricultural Standards and Testing, Beijing Academy of Agriculture and Forestry Sciences, Beijing 100097, China; lic@brcast.org.cn (C.L.); chenjy@brcast.org.cn (J.C.); cheny@brcast.org.cn (Y.C.); wangjihua@brcast.org.cn (J.W.); pingh@nercita.org.cn (H.P.); 2 Beijing Municipal Key Laboratory of Agriculture Environment Monitoring, Beijing 100097, China

**Keywords:** graphene, solid-phase extraction, polycyclic musks, water, GC–MS/MS

## Abstract

Polycyclic musks (PCMs) have recently received growing attention as emerging contaminants because of their bioaccumulation and potential ecotoxicological effects. Herein, an effective method for the determination of five PCMs in aqueous samples is presented. Reduced graphene oxide-derivatized silica (rGO@silica) particles were prepared from graphene oxide and aminosilica microparticles and characterized by scanning electron microscopy, Fourier transform infrared spectroscopy, and X-ray photoelectron spectroscopy. PCMs were preconcentrated using rGO@silica as the solid-phase extraction sorbent and quantified by gas chromatography–tandem mass spectrometry. Several experimental parameters, such as eluent, elution volume, sorbent amount, pH, and sample volume were optimized. The correlation coefficient (*R*) ranged from 0.9958 to 0.9992, while the limits of detection and quantitation for the five PCMs were 0.3–0.8 ng/L and 1.1–2.1 ng/L, respectively. Satisfactory recoveries were obtained for tap water (86.6–105.9%) and river water samples (82.9–107.1%), with relative standard deviations <10% under optimal conditions. The developed method was applied to analyze PCMs in tap and river water samples from Beijing, China. Galaxolide (HHCB) and tonalide (AHTN) were the main PCM components detected in one river water sample at concentrations of 18.7 for HHCB, and 11.7 ng/L for AHTN.

## 1. Introduction

Personal care products (PCPs) are an important class of emerging pollutants that have raised significant concerns because of their bioaccumulation ability and potential adverse effects on the ecological environment [1]. Polycyclic musks (PCMs) are a representative group of PCP compounds. PCMs are commonly used as fragrances in various consumer products, such as shampoos, body washes, and detergents [2]. PCMs can enter the water supply in effluents from municipal wastewater treatment plants. Because of their extensive use and increasing consumption worldwide in recent years, PCMs are ubiquitously detected in water environments [3,4,5,6] and even aquatic organisms [7]. PCMs have been found to bioaccumulate, with some studies suggesting that they could have ecotoxicological effects on specific organisms and cause endocrine disruption in humans [8,9,10,11]. A frequently used PCM, 1,3,4,6,7,8-hexahydro-4,6,6,7,8,8-hexamethylcyclopenta[γ]-2-benzopyran (HHCB), is among the top 50 high priority pollutants suggested by Howard and Muir, with persistence and bioaccumulation potential that require further monitoring [3,12]. As PCMs are not included in routine monitoring programs, data on their environmental impact remains insufficient [13]. To prevent adverse effects on the ecosystem, the development of a fast and sensitive method to determine these emerging organic pollutants in water is important.

Several instrumental methods have been developed for PCM determination, including high-performance liquid chromatography (HPLC) [14] and gas chromatography coupled with electron capture detection (GC–ECD), mass spectrometry (GC–MS) [15], or triple quadrupole tandem mass spectrometry (GC–MS/MS) [16,17]. GC–MS/MS can provide enhanced selectivity and sensitivity compared with conventional single quadrupole GC–MS, effectively eliminating matrix interferences. Owing to the trace PCM levels in water environments and the complexity of various water matrices, an efficient sample pretreatment step is crucial to eliminate matrix interference and concentrate the target analytes before analysis. Solid-phase extraction (SPE) is a superior and frequently used extraction technique for organic compounds in water samples. The sorbent material is a vital factor in determining the concentrating ability and recovery capacity of SPE [18]. To enrich hydrophobic organic pollutants (HOCs), reversed-phase SPE on hydrophobic or mixed-mode sorbents, such as C18-silica and *N*-vinylpyrrolidone polymer (Oasis HLB), have been commonly used [19].

Graphene is a novel two-dimensional carbonaceous material with superior chemical stability, excellent thermal stability, and an ultra-high specific surface area with a theoretical value of about 2630 m^2^/g, suggesting a high sorption capacity and great potential for use as a sorbent material [20]. Because of this, graphene and its complexes have become attractive sorbents in sample preparation procedures for a variety of analytes, such as phthalate esters [21], pesticides [22], polycyclic aromatic hydrocarbons [23]. Several pretreatment methods based on graphene have also been developed, including SPE, solid-phase microextraction (SPME), and dispersive solid-phase extraction (dSPE) [22,23,24,25,26,27,28,29]. Recently, graphene complexes were also used for the determination of synthetic musks by SPME or dSPE methods [30,31]. Despite the fact that SPE is the most frequently used extraction technique, the reported graphene materials used for PCMs by the SPE method are still limited. As an SPE sorbent, single-use graphene may lead to irreversible graphene aggregation, which could then escape the SPE cartridge under high pressure and reduce the extraction efficiency [26,32]. The immobilization of graphene on a solid support, such as silica microparticles, polymer, or steel, is an effective method to solve this problem [19]. Some studies have shown that using graphene-supported silica as a sorbent obtained excellent extraction performance for the enrichment of various analytes.

This work aimed to develop an effective and sensitive method for PCM determination in aqueous samples. The hybrid material, reduced graphene oxide-derivatized silica (rGO@silica), was synthesized and used as a new SPE sorbent for the simultaneous preconcentration of five PCMs in water. After SPE, the target analytes were quantified by GC–MS/MS. Several parameters, such as eluting solvent, sorbent amount, pH, and sample volume, were investigated. Under the optimal conditions, this novel method was successfully used for PCM determination in water samples.

## 2. Results and Discussion

### 2.1. Characterization of rGO@silica

**Figure 1:**
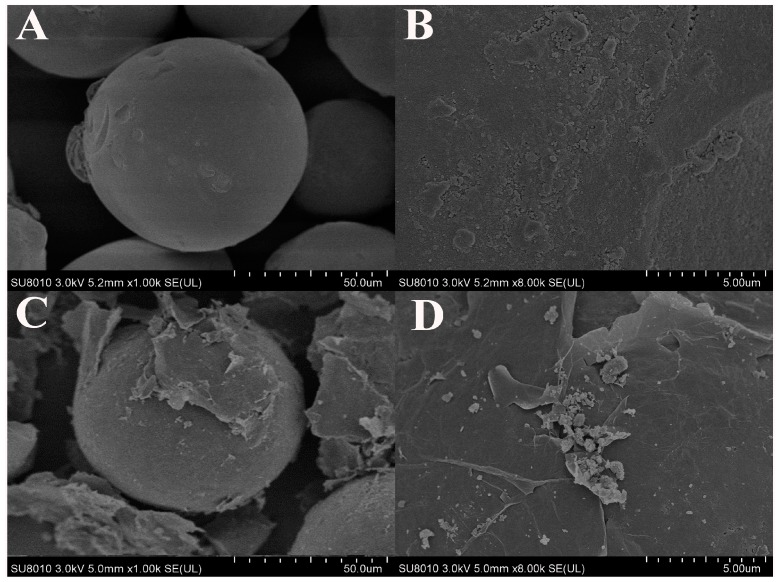
(**A**) SEM image and (**B**) high-magnification SEM image of aminosilica; (**C**) SEM image and (**D**) high-magnification SEM image of rGO@silica sorbent.

The morphology of as-prepared rGO@silica was characterized by SEM. Figure 1A,B show images of bare aminosilica microspheres. These aminosilica microparticles had a spherical shape and smooth surface. Figure 1C,D show SEM images of rGO@silica, which clearly shows that the aminosilica microspheres were tightly encapsulated by the reduced graphene oxide flakes with the typical semitransparent and crumpled sheet structure. This result suggested that the rGO layer was successfully immobilized on the surface of the aminosilica microspheres.

FT-IR spectra of aminosilica and rGO@silica are shown in Figure S1 (see Supplementary Materials). The main peaks at 798, 955, 1096, and 1640 cm^−1^ were attributed to the SiO–H bending vibration, Si–OH stretching vibration, Si–O–Si stretching vibration, and N–H bending vibration, respectively [26]. The peaks at 3450 cm^−1^ were assigned to the O–H stretching vibration. These results suggested that rGO was immobilized on the silica microsphere surface. The peak at 3450 cm^−1^ for rGO@silica was stronger than that of aminosilica because the amount of OH groups in rGO@silica was larger. These characteristic spectra were consistent with those reported by Liu et al. [26]. Furthermore, rGO@silica was characterized by X-ray photoelectron spectroscopy (XPS) (Figure S2), which showed that the carbon peak intensity of rGO@silica was much higher than that of aminosilica. This evidence further confirmed the successful rGO immobilization on the silica surface. 

### 2.2. Optimization of SPE Procedures

The sorption of PCMs on rGO@silica-packed SPE cartridge was investigated. When an aqueous solution (0.5 μg/mL) of five PCMs was passed through the cartridge, no target analytes were found in the outflow, suggesting a good retention ability for PCMs. To obtain the appropriate extraction efficiency, several experimental parameters, including elution solvent, sorbent amount, sample volume, and pH, were evaluated. The assessment was undertaken by loading rGO@silica cartridges with a standard mixture of five PCMs in water (200 mL, 100 ng/L containing 0.5% methanol). Optimization experiments were performed in duplicate and the mean values of the results were used.

#### 2.2.1. Effect of the Elution Solvent

**Figure 2:**
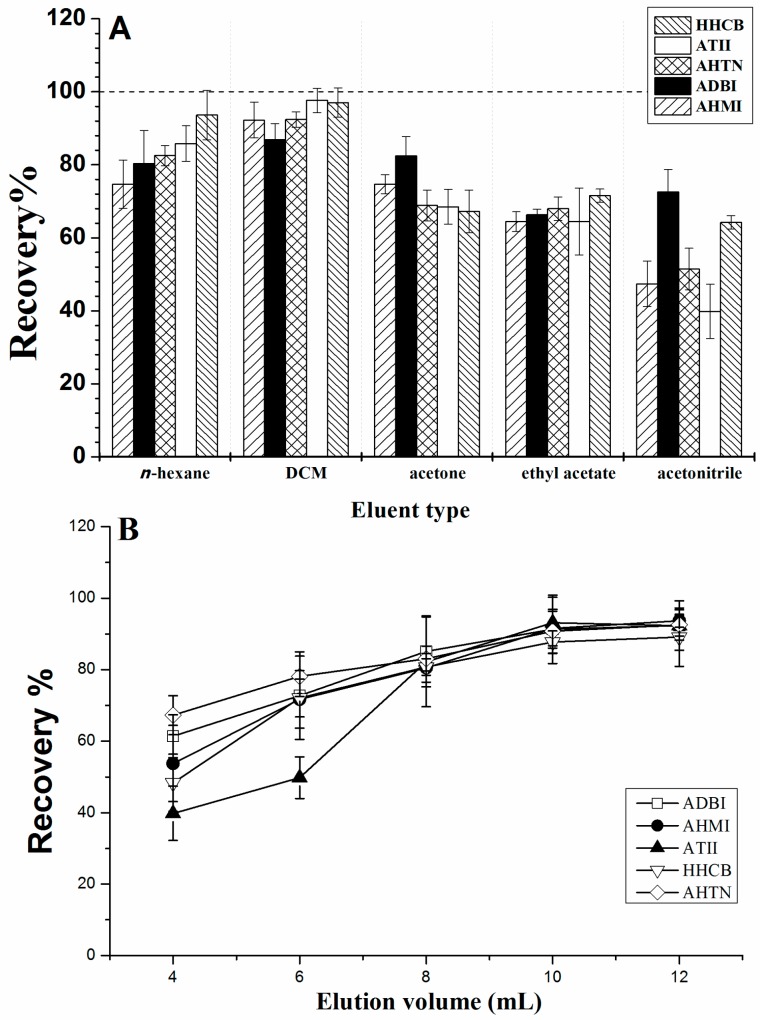
Optimization of (**A**) elution solvent and (**B**) elution volume for solid-phase extraction (SPE) of five polycyclic musks (PCMs) from water samples.

The choice of eluent is an important parameter determining the final extraction efficiency. The performances of *n*-hexane, dichloromethane (DCM), acetone, acetonitrile, and ethyl acetate as elution solvents were assessed. In each case, PCMs (20 ng of each) in aqueous solution (200 mL containing 0.5% methanol) were loaded onto the SPE cartridges, and different eluents were then passed through the cartridge. The elution efficiency of each solvent is shown in Figure 2A. DCM showed the best desorption capability for all five PCMs under the same extraction and elution conditions. Next, the volume of DCM was optimized by changing it from 4 to 12 mL over a series of tests. As shown in Figure 2B, the recoveries of the five PCMs increased with increasing DCM volume in the range 4–10 mL. Satisfactory recoveries were obtained with an eluent volume of 10 mL. Therefore, to achieve the best extraction performance, 10 mL of DCM was used in the subsequent experiments.

#### 2.2.2. Effect of the Sorbent Amount

**Figure 3:**
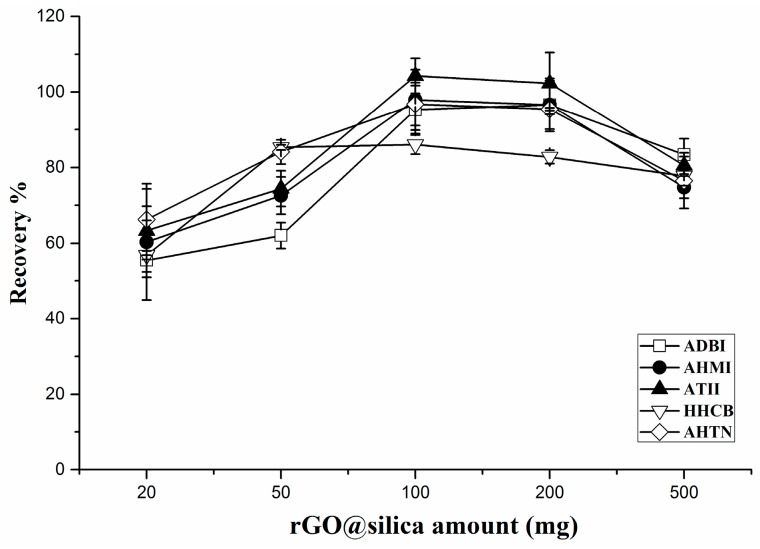
Effect of rGO@silica amount on SPE efficiency.

To ensure a sufficient analyte extraction efficiency, different amounts of rGO@silica (20–300 mg) were investigated. The same amount of PCMs (20 ng of each PCM in 200 mL of aqueous solution) was loaded to study the effect of the sorbent amount on the analyte recovery. As shown in Figure 3, as the amount of rGO@silica increased from 20 to 100 mg, the recoveries also increased. However, when the amount reached 300 mg, the recoveries slightly decreased. Therefore, 100 mg of rGO@silica was considered appropriate for the enrichment of these PCMs in water samples.

#### 2.2.3. Effect of the Sample Volume and pH

**Figure 4:**
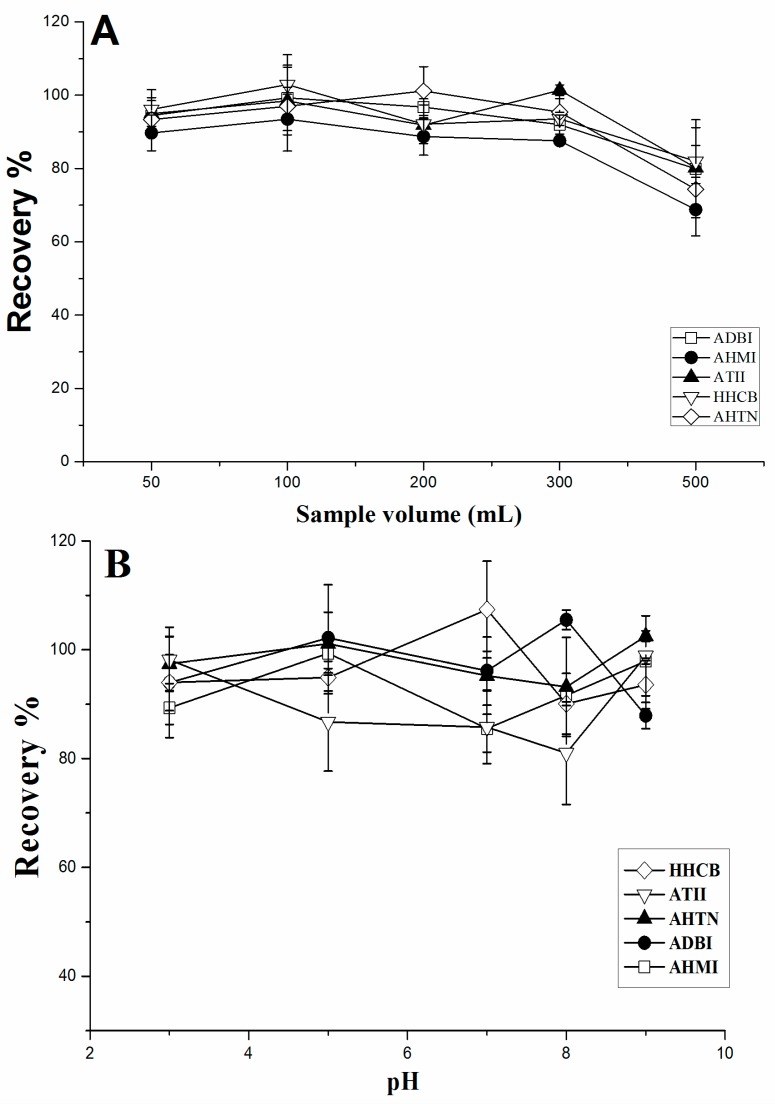
Effect of (**A**) sample volume and (**B**) solution pH on SPE efficiency.

Different volumes of aqueous solutions (50, 100, 200, 300, and 500 mL) spiked with 20 ng of each analyte were then investigated to determine the breakthrough volume, with recoveries shown in Figure 4A. Satisfactory recoveries (>85%) were obtained when the sample volume was below 300 mL. When the sample volume was increased to 500 mL, a partial sample loss or breakthrough occurred. For volumes below 500 mL, good recoveries were obtained. As tandem mass spectrometry provides enhanced sensitivity compared to conventional single quadrupole GC–MS, 200 mL of aqueous sample was able to achieve sufficient sensitivity for PCM analysis. Furthermore, a lower sample volume can reduce the extraction time. Therefore, a loading sample volume of 200 mL was used in the subsequent experiments.

The effect of the extraction pH on the recoveries was determined by adjusting the samples to various pH values ranging from 3 to 9 using 0.1 M HCl or 0.1 M NaOH. As shown in Figure 4B, no obvious variations were observed, and all five PCMs achieved good recoveries, ranging from 85.5% to 107.4%. This might be due to all PCMs being neutral under experimental conditions and their formation not being affected by different pH values. Therefore, the pH did not need to be adjusted in this study.

### 2.3. Comparison with Other Sorbents

**Figure 5:**
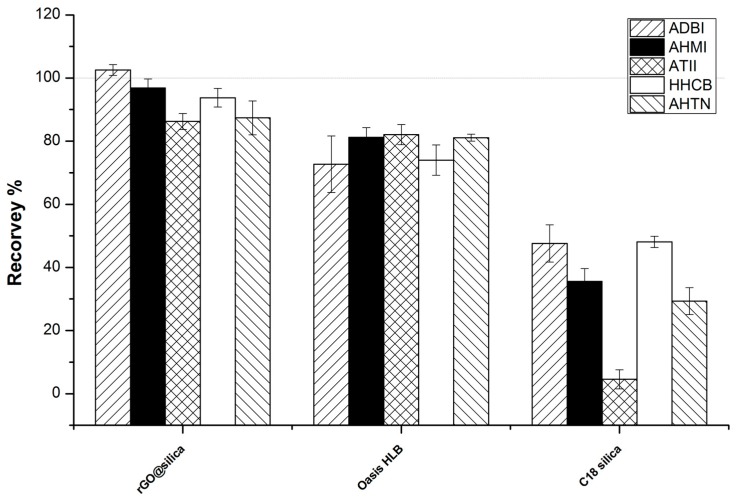
Comparison of the performance of rGO@silica with that of several other sorbents.

Because of their hydrophobic character, PCMs are commonly enriched using reverse-phase sorbents. To evaluate the enrichment capacity of rGO@silica, its performance was compared with those of conventional reserved-phase sorbent materials, including C18 silica and Oasis HLB, using the same amount (100 mg) of sorbent packed in 3 mL SPE cartridges. The cartridges were loaded with sample solutions (200 mL) spiked with the five PCMs (20 ng of each). The results are shown in Figure 5. rGO@silica yielded higher recoveries (>86%) than HLB, while C18 silica yielded the poorest recoveries (29.3–48.1%), suggesting that the sorption capacity of C18 silica was much weaker than that of rGO@silica. Therefore, rGO@silica is an excellent sorbent for PCM determination in water.

### 2.4. Method Validation

The linear regression, precision, limits of detection (LOD) and quantification (LOQ), repeatability, and recoveries of the developed method were investigated. The results are displayed in Table 1. The LOD and LOQ ranges for the five PCMs were 0.3–0.8 ng/L and 1.1–2.2 ng/L at signal-to-noise ratios of 3 and 10, respectively. The calibration curves were performed using six different concentrations (10, 20, 50, 100, 200, and 500 ng/L). A good linearity was obtained for the PCMs throughout the concentration range (*R* > 0.99).

Two kinds of water samples (tap water and river water) were considered for the application of the proposed method. Repeatability and recovery studies were conducted for both tap and river water samples. Blank water samples containing no detectable PCMs were filtered using 0.75-μm Whatman filter paper, while 200 mL samples of tap and river water were spiked with PCMs at 20, 100, and 200 ng/L in three parallel experiments. As shown in Table 2, the analyte recoveries in the fortified samples were 86.6–105.9% for tap water and 82.9–107.1% for river water. The relative standard deviations (RSDs) for all tap and river samples were below 10%. Figure S3 shows a typical total ion chromatogram (TIC) and multiple reaction monitoring (MRM) chromatogram of the tap water sample spiked with PCMs at 100 ng/L.

### 2.5. Application to Real Samples

The method was used to analyze five PCMs in three tap water and three river water samples from Beijing. The results showed that no PCMs were detected in the tap water from Beijing, while HHCB and tonalide (AHTN) were found in one of the river water samples, with concentrations of 18.7 and 11.7 ng/L for HHCB and AHTN, respectively.

## 3. Materials and Methods

### 3.1. Reagents and Materials

Standard solutions of five polycyclic musks, namely, celestolide (ADBI), phantolide (AHMI), traseolide (ATII), galaxolide (HHCB), and tonalide (AHTN), with concentrations of 10 mg/L were obtained from Dr. Ehrenstorfer (Augsburg, Germany). The internal standard, ^13^C_6_-labeled hexachlorobenzene (^13^C_6_-HCB, 100 mg/L), was obtained from Cambridge Isotope Laboratories (Andover, MA, USA). Pesticide-grade *n*-hexane, dichloromethane (DCM), acetone, and chromatography-grade ethyl acetate were supplied by Fisher Scientific (J.T. Baker, Pittsburgh, PA, USA). *N*,*N*′-Dicyclohexylcarbodiimide (DCC) was obtained from Alfa Aesar (Ward Hill, MA, USA). Hydrazine hydrate (85%) was purchased from Sinopharm Chemical Reagent Co., Ltd. (Shanghai, China). Analytical-grade *N*,*N*-dimethylformamide (DMF) was obtained from J&K Scientific Ltd. (Beijing, China).

Graphene oxides (GOs) were purchased from Nanjing XFNANO Materials Tech (Nanjing, China). Aminosilica and C18 silica were obtained from Agilent (Santa Clara, CA, USA). *N*-Vinylpyrrolidone polymeric cartridges (Oasis HLB) were obtained from Waters (Milford, MA, USA).

### 3.2. Preparation and Characterization of rGO@silica

GO@silica hybrids were prepared by linking the GO carboxy groups with amino groups on spherical aminosilica microparticles, similar to the procedure reported by Liu et al. [21]. To summarize, GO (100 mg) was dispersed in DMF (150 mL) by sonication for 30 min. Next, aminosilica (1 g) and DCC (100 mg) were added, and the mixture was stirred at 50 °C for 24 h. The solid product was washed with water and methanol several times to remove unbound GO. The collected GO@silica was dried at 60 °C for 12 h. rGO@silica was obtained by reducing GO@silica with hydrazine. Briefly, GO@silica (1 g) and hydrazine hydrate (0.5 mL, 85%) were added to water (50 mL), and the mixture was heated at 95 °C for 2 h. The solid product was filtered, washed with ultrapure water and methanol, and dried at 60 °C for 12 h.

The surface morphology of the materials was characterized using field-emission scanning electron microscopy (FESEM; Hitachi-S-4800, Hitachi, Tokyo, Japan). X-ray photoelectron spectroscopy (XPS) was performed on a Thermo Scientific ESCALAB 250Xi XPS system (Waltham, MA, USA). The surface functional groups were analyzed by Fourier transform infrared spectroscopy (FT-IR) using a Nicolet 6700 FT-IR spectrometer (Thermo Nicolet, Madison, WI, USA).

### 3.3. Analytical Procedure

SPE was performed on a SPE Vacuum Manifold apparatus (Sigma-Aldrich, St. Louis, MO, USA) equipped with a V700 vacuum pump (Buchi, Flawil, Switzerland). To prepare the SPE cartridges, rGO@silica powder (100 mg) was packed into a 3 mL polypropylene column with an upper and a lower frit to avoid sorbent loss. For PCM enrichment, the cartridge was preconditioned with methanol (10 mL) and water (10 mL). The aqueous sample solution containing 0.5% methanol was then passed through the cartridge at a flow rate of about 5 mL/min. After extraction, the cartridge was washed with a 5% methanol aqueous solution (10 mL) and then was dried under vacuum for 5 min to remove residual water. The retained analytes were then eluted with DCM (8 mL). The effluent was evaporated to dryness under a stream of nitrogen and reconstituted in *n*-hexane (1 mL). Before GC–MS/MS analysis, the extracts were spiked with the internal standard (100 ng).

The GC–MS/MS system consisted of an Agilent 7890B GC instrument equipped with an Agilent 7693B autosampler and an Agilent 7000C triple quadrupole system (Agilent Technologies, Palo Alto, CA, USA). GC separation was performed using two identical Agilent J&W HP—5 ms UI capillary columns (15 m × 0.25 mm I.D., 0.25-μm film thickness) connected through an auxiliary programmable control module. Backflushing was performed to shorten the analysis time and reduce the system maintenance. The injector temperature was held at 280 °C for the entire run. Helium (99.999%) was used as the carrier gas at a constant flow rate of 1 mL/min. The oven temperature program was set as follows: initial temperature, 60 °C for 1 min; increased to 170 °C at 40 °C/min; then increased to 230 °C at 10 °C/min; then ramped to 280 °C at 30 °C/min; and finally held at 280 °C for 1 min. The total run time was 12.4 min, plus an additional 3 min for backflushing at 300 °C.

The multiple reaction monitoring (MRM) mode was used for monitoring and for the confirmation analysis. The manifold temperature was maintained at 230 °C. Quad MS1 and MS2 temperatures were set to 150 °C. The flow rate of the collision gas (N_2_) was set to 1.5 mL/min. Two MS/MS transitions were used for each analyte, with the sensitivity optimized using collision energy experiments. MS/MS transitions, collision energies, chromatographic retention times, and molecular and chemical information for each PCM are shown in Table S1 (see Supplementary Materials).

## 4. Conclusions

In this study, a simple and novel method was developed for the enrichment and determination of five PCMs in environmental water samples. The rGO@silica was synthesized by graphene oxide and aminosilica as SPE material, showing high adsorption capacity for PCMs. GC–MS/MS was employed for the quantification. Under the optimized condition, satisfactory recoveries, low LODs, and good repeatability were observed for both tap and river water samples. The LOD and LOQ for the five PCMs were 0.3–0.8 ng/L and 1.1–2.1 ng/L, respectively. The recoveries were 86.6–105.9% for tap water and 82.9–107.1% for river water samples, with relative standard deviations <10%. The obtained results indicated that rGO@silica has great potential in the separation and preconcentration of PCMs from water samples. Additionally, this developed method was used to analyze PCMs in tap and river water samples from Beijing, China. HHCB and AHTN were the main PCM components detected, at concentrations of 18.7 ng/L for HHCB, and 11.7 ng/L for AHTN, in one river water sample.

## Figures and Tables

**Table 1:** Analytical parameters for PCM determination using GC–MS/MS.

Analyte	Linear Range (ng/L)	*R*	LOD (ng/L)	LOQ (ng/L)
ADBI	10–500	0.9992	0.5	1.5
AHMI	10–500	0.9978	0.3	1.1
ATII	10–500	0.9958	0.8	2.1
HHCB	10–500	0.9976	0.6	1.4
AHTN	10–500	0.9977	0.5	1.2

**Table 2:** Recovery studies on tap water and river water samples containing PCMs.

		Tap Water Sample		River Water Sample	
Analyte	Spiked Levels (ng/L)	Recovery (%)	RSD (%)	Recovery (%)	RSD (%)
ADBI	50	91.3	5.2	88.6	5.7
	100	102.4	6.1	102.3	3.5
	200	97.8	4.5	82.9	3.9
AHMI	50	89.4	1.9	87.1	2.6
	100	99.2	2.3	97.1	3.8
	200	92.3	0.8	93.1	5.2
ATII	50	99.1	2.1	96.9	5.8
	100	98.8	2.4	101.1	3.3
	200	89.6	3.4	85.3	5.9
HHCB	50	96.9	2.7	107.1	2.5
	100	93.1	5.2	106.3	3.3
	200	86.6	1.7	103.9	3.1
AHTN	50	93.3	5.9	99.7	5.7
	100	95.6	2.9	96.4	4.3
	200	105.9	5.5	84.5	3.1

## Supplementary Materials

The following are available online, Table S1: Chemical information and MS/MS parameters of five PCMs and the internal standard. Figure S1: FT–IR spectra of aminosilica and prepared rGO@silica. Figure S2: Overview of XPS spectra for aminosilica and prepared rGO@silica. Figure S3: Typical chromatograms of tap water spiked with PCMs (100 ng/L): (A) TIC mode; (B) MRM mode.
