# Supplementary material for: Graphene-Derivatized Silica Composite as Solid-Phase Extraction Sorbent Combined with GC–MS/MS for the Determination of Polycyclic Musks in Aqueous Samples
Source: Molecules. 2018 Feb 2;23(2):318. doi: 10.3390/molecules23020318 (PMC6017199; doi:10.3390/molecules23020318)
Supplement: Supplementary file 1 [file molecules-23-00318-s001.pdf]

Supplementary Materials to:

Graphene-derivatized silica composite as solid phase extraction sorbent combined with GC-MS/MS for determination of polycyclic musks in aqueous samples

Cheng Li [1,2], Jiayi Chen [1,2], Yan Chen [1,2], Jihua Wang [1], Hua Ping [1] and Anxiang Lu [1,2,*]

[1] Beijing Research Center for Agricultural Standards and Testing, Beijing Academy of Agriculture and Forestry Sciences, Beijing 100097, China; lic@brcast.org.cn (C.L.); chenjy@brcast.org.cn (J.C.); cheny@brcast.org.cn (Y.C.); wangjihua@brcast.org.cn (J.W.); pingh@nercita.org.cn (H.P.)

[2] Beijing Municipal Key Laboratory of Agriculture Environment Monitoring, Beijing 100097, China

* Correspondence: anxiang_lu@hotmail.com; Tel.: +86-10-51503057

1. Supporting Tables

Table S1. Chemical information and MS/MS parameters of five PCMs and the internal standard.

Compound Name	CAS Number	Molecular Formula	Molecular Weight	Molecular Structure	Retention time (min)	Transition 1	CE^{a} 1 (eV)	Transition 2	CE 2 (eV)
ADBI	13171-00-1	C17H24O	244.4	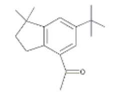	7.49	244.0 -> 229.2	10	229.0 -> 173.1	5
AHMI	15323-35-0	C17H24O	244.4	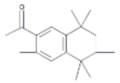	7.84	244.0 -> 229.2	10	229.0 -> 187.2	30
ATII	68140-48-7	C18H26O	258.4	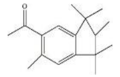	8.56	258.0 -> 215.0	10	215.2 -> 173.2	5
HHCB	1222-05-5	C18H26O	258.3	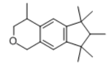	8.61	258.2 -> 243.2	10	243.2 -> 213.2	10
AHTN	21145-77-7	C18H26O	258.4	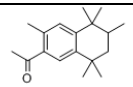	8.69	258.2 -> 243.2	5	243.2 -> 187.2	5
$^{13}C_{6}$-HCB （Internal standard）	93952-14-8	C6Cl6	290.7	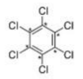	7.61	289.7 -> 254.8	25	289.7 -> 219.9	30

Figure S1

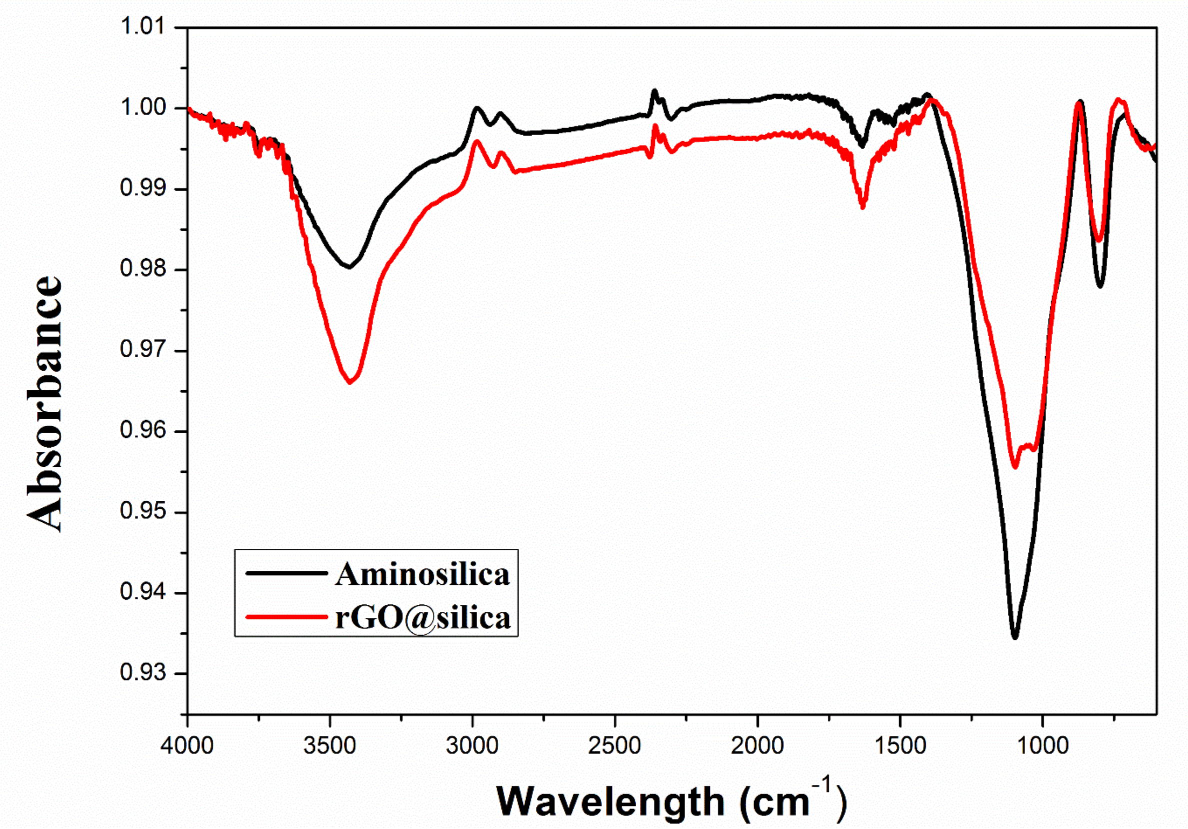

Fig. S1 FT-IR spectra of aminosilica and prepared rGO@silica.

Figure S2

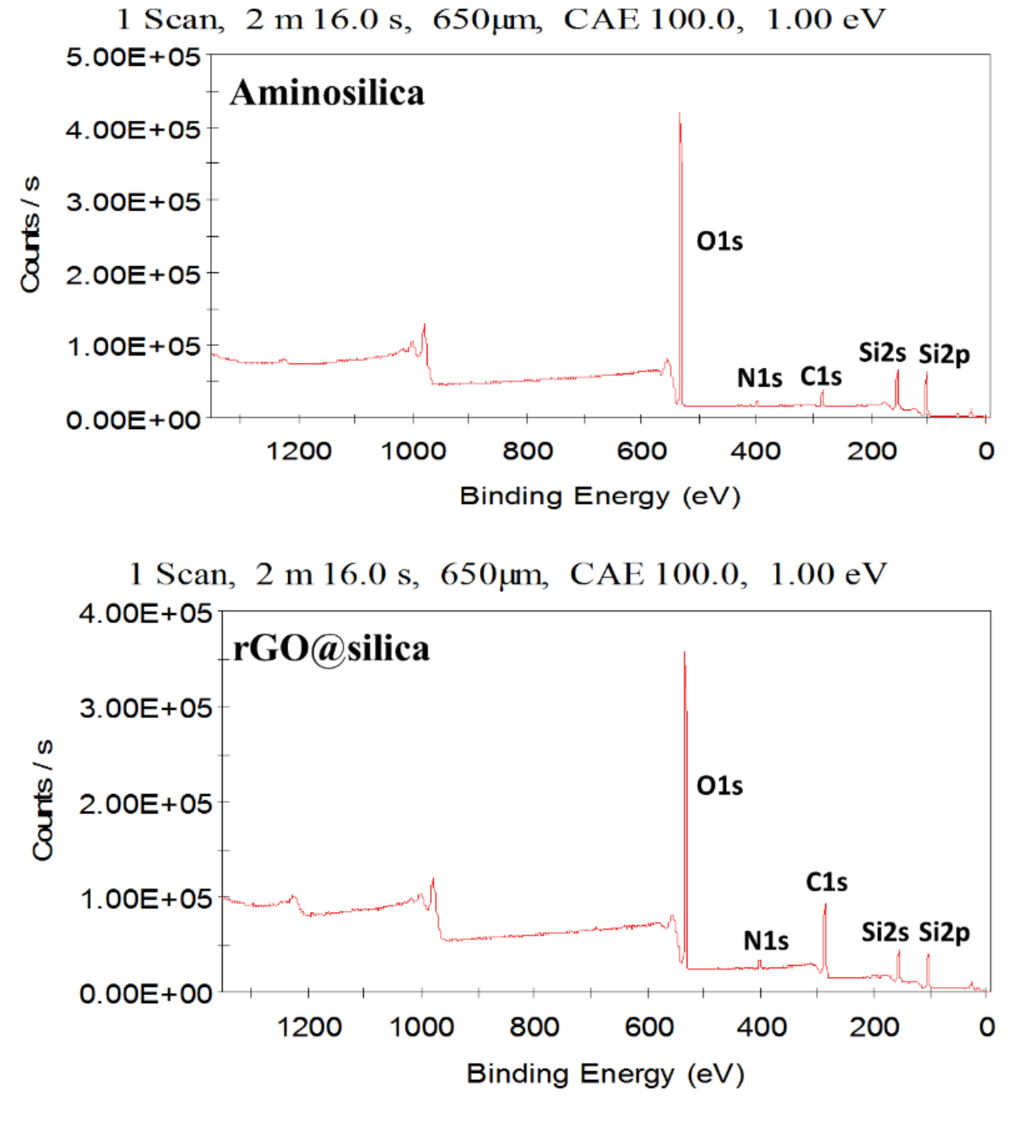

Fig. S2 The overview of XPS spectra for aminosilica and prepared rGO@silica

Figure S3

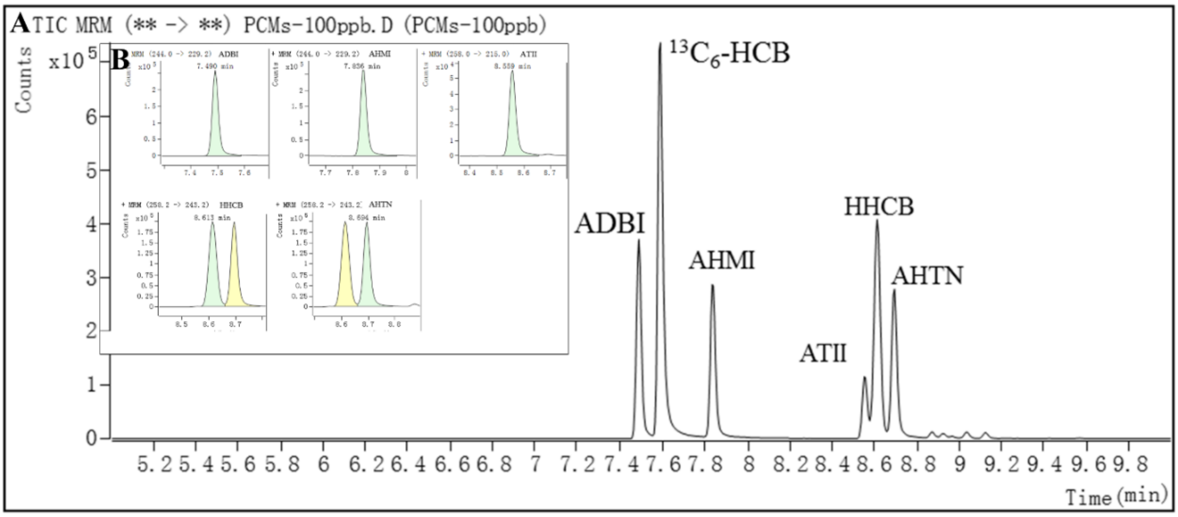

Figure S2. Typical chromatograms of tap water spiked with PCMs (100 ng/L): (A) TIC mode; (B) MRM mode.
